# Preservation of Nociceptor Intraepidermal Nerve Fibres in Diabetic Neuropathic Pain

**DOI:** 10.1155/jdr/4561974

**Published:** 2026-09-07

**Authors:** Lisa A. Lione, Lydia D. Hardowar, Michael T. Lanigan, Jessica Bates, William Blackstone-Whines, Harry Jones, Richard P. Hulse

**Affiliations:** ^1^ School of Health, Medicine and Life Sciences, University of Hertfordshire, Hatfield, UK, herts.ac.uk; ^2^ School of Science and Technology, Nottingham Trent University, Nottingham, UK, ntu.ac.uk

**Keywords:** CGRP, diabetes, inflammation, neuropathic pain, nociceptor, pain

## Abstract

Peripheral painful sensory neuropathy represents a prevalent and disabling complication of Type 1 and Type 2 diabetes. Unfortunately, despite the clinical need for improved analgesic approaches, clinicians remain inadequately provisioned to treat these patients as many analgesics are ineffective or initiate undesirable health‐impacting side effects. During diabetes, the viability of the peripheral sensory nervous system is vulnerable, depicted by sensory neurodegeneration and neuropathic pain. However, it remains undefined how nociceptor intraepidermal fibre innervations are affected in patients and rodent models. Here, rodent models of chemically induced Type 1 and dietary induced prediabetes/Type 2 diabetic peripheral sensory neuropathy were utilised to investigate the impact upon nociceptor degeneration in painful diabetic neuropathy. Streptozotocin‐induced Type 1 diabetes as well as 42% and 60% high‐fat diets caused mechanical allodynia when compared with age‐matched controls. Furthermore, dietary interventions led to a sustained hypersensitivity to thermal stimuli and a transient mechanical allodynia that was observed in the early phase, which later resolved. Histological analysis of plantar skin of the hind limbs demonstrated a significant reduction in total intraepidermal nerve fibre (IENF) density (PGP9.5 labelled), indicating sensory neurodegeneration in Type 1 and prediabetes/Type 2 diabetic rodent models. However, CGRP‐positive nociceptor IENFs remain preserved, pointing to differential vulnerability among sensory fibre subtypes. Additionally, in Type 1, there was an increased presence of Langerhan cells, whereas this was not presented in the dietary induced models. This provides mechanistic insight into painful diabetic neuropathy and provides fundamental understanding that will support improved treatment of diabetic sensory neuropathy.

**What is already known?**

Painful diabetic neuropathy is common in Type 1 and Type 2 diabetes. Analgesics are often ineffective or have adverse effects. Sensory neurodegeneration underlies neuropathic pain, but nociceptor fibre changes are undefined.

**What this study has found?**

Type 1 and high‐fat diet rodent models caused mechanical allodynia and thermal hypersensitivity which occurred in dietary model. Total intraepidermal nerve fibres decreased, with nociceptor CGRP fibres preserved. Langerhans cells increased in only the Type 1 model.

**What are the implications?**

This demonstrates selective sensory nerve fibre vulnerability, guiding mechanistic understanding and improved treatments for painful diabetic neuropathy.

## 1. Introduction

Diabetic peripheral sensory neuropathy (DPSN) is one of the most common complications of diabetes mellitus, affecting approximately 50% of individuals with either Type 1 or Type 2 diabetes [1]. DPSN presents as a distal symmetrical polyneuropathy manifesting with pain, tingling, numbness, typically in a ‘glove and stocking’ distribution. There is a positive correlation between poor glycaemic control, the severity of DPSN and risk and intensity of neuropathic pain [2]. The progression of DPSN significantly impairs patients′ mobility and negatively affects sleep and mental wellbeing [3] whilst also contributing to increased risk of foot ulceration and subsequent amputations [4]. Notably, current treatments for painful DPSN, including anticonvulsants, antidepressants and topical agents, offer only partial symptomatic relief [5, 6] and are often accompanied by dose‐limiting side effects that limit their long‐term use [7]. Therefore, there is a major unmet clinical need for therapies that target the underlying mechanisms of nerve dysfunction to provide robust painkilling intervention.

Clinically, the diagnosis and monitoring of DPSN involve a combination of patient‐reported symptoms, quantitative sensory testing (QST) and electrophysiological measurements. In addition, sensory neurodegeneration is demonstrated by intraepidermal small nerve fibre (IENF) loss [8], which correlates strongly with clinical symptoms, pain severity and sensory deficits in DPSN patients, making it a valuable tool in both clinical diagnosis and therapeutic trials. This allows focus upon small fibre innervation at the dermal–epidermal border, which is highly susceptible to damage in diabetic patients [9]. However, this predominantly relies upon a single marker, protein gene Product 9.5 (PGP9.5), for IENF identification that represents total sensory nerve fibre skin innervation patterns across the sensory neuronal population. This does not consider the distinction between differing sensory neuronal clustered populations and principally the impact upon the small fibre or nociceptor subtype is underrepresented. Langerhans cells (LCs) are resident macrophages closely associated with IENFs in the epidermis that are identified by PGP9.5 [10]. Understanding the cellular mechanisms that underlie this sensory neurological complication in diabetes is essential for the development of more effective treatments. Rodent models of diabetes, particularly those incorporating a single injection of streptozotocin (STZ) [11, 12] or dietary induction of metabolic disease [13], provide valuable insight into the early stages of neuropathy. Unlike the widely studied STZ chemically induced model that mimics Type 1 diabetes, high‐fat diet (HFD) models reflect the gradual metabolic dysfunction associated with Type 2 diabetes, including insulin resistance, hyperglycaemia, dyslipidaemia and low‐grade inflammation [14]. These features are especially relevant given that over 90% of diabetic patients have Type 2 diabetes [15]. Additionally, pain behaviours in rodent models of diabetes such as mechanical allodynia and thermal hyperalgesia can be quantified using methods that parallel clinical QST, enabling translational assessment of potential therapies.

In this study, a rodent model of Type 1 diabetes and a dietary induced model representing a rodent model of induced metabolic disturbance, prediabetes and Type 2 diabetes was adopted to investigate small fibre innervation density. We assessed nociceptive behavioural phenotypes to mechanical and heat stimuli and evaluated intraepidermal nerve fibre density (IENFD) and LC abundance. This multifaceted approach mirrored clinical practice by integrating behavioural, structural and immune markers of neuropathy. Here, we demonstrate nociceptor subsets underlie painful peripheral sensory neuropathy allowing improved understanding of the principle mechanisms involved and to allow discovery of new targeted treatments aimed at halting or reversing sensory neurodegeneration and diabetic neuropathic pain.

## 2. Materials and Methods

### 2.1. Ethical Approval and Animals Used

All procedures followed the Animals (Scientific Procedures) Act 1986 and EU Directive 2010/63/EU, with approval from the University of Hertfordshire and Nottingham Trent University Ethics Boards and by following the ARRIVE guidelines. Animal numbers and exposure durations were minimised in line with NC3Rs. Adult male and female C57/BL6J mice (*n* = 57; male = 39, female = 18) and 30 male Wistar Han ISG rats (Charles River, United Kingdom) were used. Animals were housed in enriched environments on a 12:12 h light–dark cycle, with ad libitum access to standard chow and water. For STZ studies, water was provided in two 1 L bottles per cage. For 48 h after STZ administration (Day 0, 1 and 2), 2% sucrose solution was provided in one of the 1 L bottles to avoid hypoglycaemia caused by STZ administration. Water intake measurements were recorded for the total liquid intake from 1 week before STZ administration and as required until the end of the study. Water intake was calculated as the change in weight (g) of each food hopper and both water bottle recorded at approximately 9:00 AM each day. A period of food restriction was performed prior to fasted blood glucose measurement, with this period occurring in the morning being a minimum of 2 h and no longer than 4 h.

### 2.2. Rodent Models of Diabetic Sensory Neuropathy

For Type 1 diabetic neuropathy, rats received a single ip injection of 55 mg/kg STZ (Sigma‐Aldrich, S0130, *n* = 18; 10 mL/kg in 20 mM citrate buffer, pH 4.1) or 20 mM citrate buffer as control (*n* = 12). For 48 h following STZ/control dosing, a 2% sucrose solution was provided in one water bottle, selected at random either the left or right bottle, to avoid hypoglycaemia caused by STZ. Food (5LF2 10% protein LabDiet changed to 5LF5 22% protein LabDiet on day of STZ dosing) and drinking water were provided ad libitum except during von Frey behavioural testing periods. Fasted blood glucose was measured by tail‐tip pricks using a GlucoRx Nexus blood glucose monitor at baseline and days 7, 21, 35 and 45 postdosing to confirm onset and maintenance of hyperglycaemia (>16 mmol/L) [16]. To monitor animal welfare bodyweight, measurements were taken daily prior to STZ/control dosing (baseline) and daily throughout the study. On Day 45, animals were culled via Schedule 1 procedure. Plantar hind paw skin was collected and fixed in 4% PFA (10% neutral buffered formalin), removed after 24 h, rinsed in PBS and placed in 30% sucrose solution prior to embedding in OCT. These cryoprotected tissues were stored at −80°C until tissue preparation and processing.

For diet‐induced models, after baseline readings, cages were maintained either on standard chow or switched to 42% (by kcal TD.88137 Envigo, United States fat diet) or 60% (by kcal TD.06414 Envigo, United States fat diet) HFDs. Weekly cage cleaning and daily water replenishment were conducted. Fasted blood glucose was measured from tail‐tip pricks. At study end, animals were culled via Schedule 1 procedure. Hind paw skin was collected, which was placed in 4% PFA (10% neutral buffered formalin), removed after 24 h, rinsed in PBS and placed in 30% sucrose solution prior to embedding in OCT. These cryoprotected tissues were stored at −80°C until tissue preparation and processing.

### 2.3. Nociceptive Behavioural Assays

Mice and rats were habituated to experimenter handling and experimental environment prior to the onset of nociceptive behavioural testing. Nociceptive behavioural assays, von Frey and Hargreaves tests, were performed to evaluate nociceptive behavioural phenotypes and as previously described [17, 18].

### 2.4. Mechanical Nociceptive Behavioural Assay

For von Frey testing, the rats or mice were placed within clear modular holder Perspex cages (Bioseb, United States). The enclosures were placed on top of mesh flooring to enable access for application of von Frey monofilaments (Bioseb, United States) to the medial paw plantar surface of the rat or mouse hind paw. Testing was performed after an initial 15–20 min acclimatisation period.

For rats, a range of von Frey monofilaments with increasing force (0.4–26 g) was applied to the hind paw. The up‐down method was used to establish the paw withdrawal threshold (PWT) by applying filaments of increasing or decreasing force depending on the previous response [19, 20]. Monofilaments were placed perpendicular under the hind foot pad of the animal and applied to the plantar skin until it buckled, delivering a uniform and constant force for a set period of time (2–8 s). An immediate sharp withdrawal response from the stimulus (or flinching) or paw licking was considered to represent a positive response.

For mice, a range of von Frey monofilaments with increasing force (0.16–2 g) was applied to the hind paw. Each von Frey was applied to a hind paw for a minimum interval of 5 min; mechanical nociceptive withdrawals were scored for both left and right paws to allow mechanical nociceptive withdrawal thresholds to be determined [18].

### 2.5. Heat Nociceptive Behavioural Assay

For Hargreaves testing, the mice were placed within clear Perspex enclosures on top of a transparent framed glass pane. An infrared generating emitter/detector was placed beneath the glass panel positioned under the hind paw. When the animal withdraws the paw away from the stimulus, the automatic timer stops and the latency of the paw heat nociceptive withdrawal was recorded [18]. Hargreaves laser intensity was set at baseline acquisition with typically setting at 45.

### 2.6. Immunohistochemistry (IHC)

Skin samples were dehydrated in ethanol (70%, 90% and 100%), cleared overnight in xylene and embedded in paraffin. Paraffin sections (10 *μ*m) were cut using a microtome and mounted on Epredia Polysine slides. For cryosectioning, frozen tissue was mounted in OCT. Cryosections (10 *μ*m) were cut and mounted on SuperFrost Plus slides, dried for 30 min and stored at –20°C. Paraffin‐embedded sections were deparaffinised and rehydrated using xylene and graded ethanol, followed by TBS‐Triton washes. Heat‐induced antigen retrieval was performed using citrate buffer (pH 6.0) in a pressure cooker at 110°C for 3 min. All slides were cooled on ice, washed and incubated with 0.3% H_2_O_2_ for peroxidase blocking, followed by blocking in bovine serum albumin (5%). Primary antibodies against PGP 9.5 (rabbit Abcam, Cambridge, United Kingdom, ab108986, 1:300 diluted with TBS + 0.025% triton x‐100) and CGRP (rabbit, Abcam, Cambridge, United Kingdom, ab47027, 1:400 diluted with TBS + 0.025% triton x‐100) were applied and incubated overnight at 4°C. After TBS‐Triton washes, antirabbit biotinylated secondary antibody (Jackson Laboratories, donkey, 711‐067‐003) and streptavidin‐HRP (Abcam, Cambridge United Kingdom, ab64269) were added sequentially, followed by DAB substrate for colour development. Slides were dehydrated, cleared in xylene and coverslipped with DPX. IHC reagents were from the Abcam HRP/DAB IHC kit (ab64261). Slides were imaged at 40x magnification on a Nikon eclipse TE2000‐U microscope. In some instances, antirabbit secondary Alexa Fluor 488 (ab150073) and DAPI were applied. Subsequently, vectorshield (VWR, H1000) was added to slides with coverslips applied and were protected from light until imaged. Plantar skin sections were imaged as z‐stacks (1024 × 1024 pixels) using Leica SP5 confocal at ×20, with three to five non‐sequential sections acquired per animal. Skin sections were only utilised for further analytical observations if the region of interest, epidermal–dermal border, was undamaged and/or free from damage.

### 2.7. Data Acquisition and Analysis

Images were analysed using ImageJ. From each animal, three stained slices were assessed. Nerve fibres and LCs were counted in five random areas per section (15 measurements/animal), with data presented as averages per animal. Epidermal section length was measured to calculate intraepidermal nerve fibre (IENF) and LC linear densities. Measures of IENF were determined by counting IENF when they cross the dermal–epidermal border as individual units, with postcrossing branches counted as one, submembrane splits as two and fragments or fibres that fail to cross the basement membrane excluded entirely and normalised to length of section per mm [21]. Strict criteria were used for counting fibres crossing the dermal–epidermal junction. Nociceptive withdrawal measures were recorded for left and right hind paws, with averages represented in presented data per animal. In this study we present robust data that encapsulated a differing models of metabolic induced sensory neuropathy, with sample sizing determined for quantitative nociceptive behavioural experiments, calculated in G∗Power using a priori power analysis, with *α* set at 0.05 and power at 80% (*β* = 0.2). Expected effect sizes and variance are derived from our previous datasets. All samples were anonymised using opaque tape and randomised numbering during analysis. Statistical analysis was conducted using GraphPad Prism 9. Data are presented as mean ± SEM. Independent *t*‐tests, one‐way ANOVA and two‐way ANOVA (with Bonferroni post hoc) were used as appropriate. Correlations between nerve parameters and pain data were analysed using Pearson correlation.

## 3. Results

**Figure 1:**
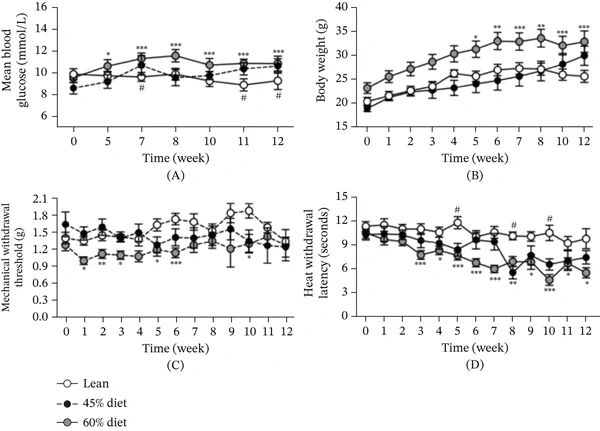
Dietary induced neuropathic pain. Male and female C57 BI6 mice were fed either control chow male (lean; *n* = 34), 42% fat diet (*n* = 12) or 60% fat diet (*n* = 34) for 12 weeks, with nociceptive (weekly) and metabolic (body weight weekly, blood glucose at designated intervals) measures performed regularly. (A) Fasted blood glucose and (B) body weight were recorded regularly during the study, with 60% HFD dietary intervention inducing elevations in fasted blood glucose and body weight. (C) Mechanical nociceptive withdrawal threshold were reduced initially following 60%HFD dietary intervention that later resolved compared with lean control group. (D) Heat nociceptive withdrawal latency was measured using Hargreaves test demonstrating a robust reduction following 60% HFD dietary intervention, with 42% HFD inducing partial hypersensitivity (two‐way ANOVA with mixed effects and multiple comparisons,  ^∗^ *p* < 0.05,  ^∗∗^ *p* < 0.01 and  ^∗∗∗^ *p* < 0.001 = lean vs. 60% diet and # *p* < 0.05 = lean vs. 42% diet).

**Figure 2:**
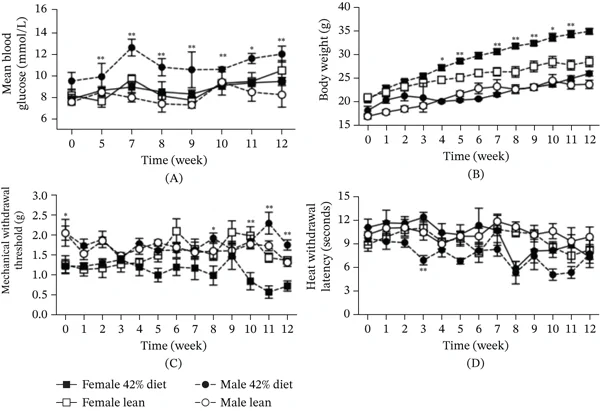
42% Fat dietary induced neuropathic pain. Male and female C57 BI6 mice were fed either control chow (male lean; *n* = 28, female lean; *n* = 6) or 42% fat diet (male 42%; *n* = 6, female 42%; *n* = 6). (A) Fasted blood glucose and (B) body weight were recorded regularly during the study, with 42% HFD dietary intervention in male mice inducing elevations in fasted blood glucose and increased body weight when compared with females. (C) Mechanical nociceptive withdrawal threshold was reduced following 42% HFD dietary intervention in females compared with male 42% group. (D) Heat nociceptive withdrawal latency was measured using Hargreaves test demonstrating minimal differences between sexes with 42% HFD intervention (two‐way ANOVA with mixed effects and multiple comparisons,  ^∗^ *p* < 0.05,  ^∗∗^ *p* < 0.01 and  ^∗∗∗^ *p* < 0.001 and # *p* < 0.05, comparisons made between male and female).

**Figure 3:**
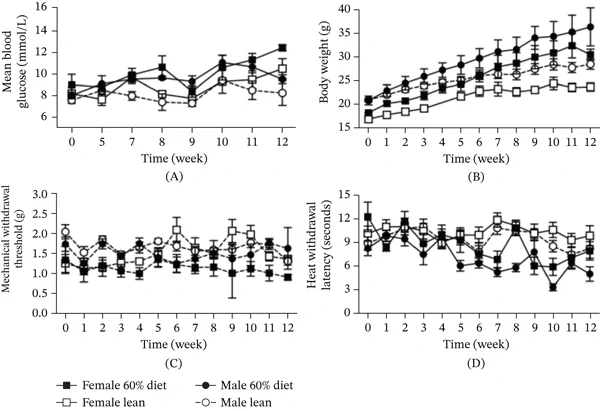
60% Fat dietary induced neuropathic pain in females. Male and female C57 BI6 mice were fed either control chow (male lean; *n* = 28, lean; *n* = 6) or 60% fat diet (male 60%; *n* = 28, female 60%; *n* = 6). (A) Fasted blood glucose and (B) body weight were recorded regularly during the study. (C) Mechanical nociceptive withdrawal threshold with no alterations in between male or females were observed (D) Heat nociceptive withdrawal latency was measured using Hargreaves test demonstrating no alteration between male and female groups (two‐way ANOVA with mixed effects and multiple comparisons,  ^∗^ *p* < 0.05 and  ^∗∗^ *p* < 0.01, comparisons made between male and female).

Peripheral sensory neuropathy was explored in differing rodent models of diabetes including in an established Type 1 rat model of STZ‐induced diabetic sensory peripheral neuropathy [22] and dietary induced peripheral sensory neuropathy. In the HFD‐induced sensory neuropathy model, both male and female mice were fed either a control chow, 42% HFD or 60% HFD for 12 weeks. Fasted blood glucose levels (Figure 1A) and body weight (Figure 1B) were monitored, with 60% HFD demonstrating increased weight compared with lean control diet (Figure 1B). Elevated blood glucose levels were also observed (Figure 1A). The 60% HFD led to a transient mechanical allodynia that developed within the early weeks post‐alteration of diet, which resolved prior to the end of the study (Figure 1C). Whereas heat hyperalgesia became increasingly apparent and established over the 12 weeks, with 42% HFD also inducing heat hypersensitivity (Figure 1D). Metabolic features were consistent across male mice (Figure S1A,B) and female (Figure S2A,B) mice from both HFD groups. Male showed initial reductions in mechanical withdrawal thresholds; however, this was resolved after 7 weeks (Figure S1C). The 60% HFD induced heat hypersensitivity in male mice (Figure S1D). In female mice, mechanical allodynia did not establish until Week 10 of the study (Figure S2C), whereas heat hypersensitivity appeared between Weeks 7–10 and subsequently resolved returning back towards baseline (Figure S2D). Comparisons between males and females on the 42% fat calorie dietary intervention demonstrated alterations in blood glucose (Figure 2A) with differences in increased body weight progression (Figure 2B). Furthermore, female mice on 42% diet had pronounced mechanical hypersensitivity (Figure 2C), whereas there were no changes in male mice. There were no differences in nociceptive behavioural withdrawal latency to heat in either male or female mice (Figure 2D). Progression of metabolic induced peripheral sensory neuropathy was compared between males and females on the 60% fat calorie dietary intervention. There were no alterations in blood glucose (Figure 3A), in increased body weight progression (Figure 3B), mechanical hypersensitivity (Figure 3C) or nociceptive behavioural withdrawal latency to heat (Figure 3D) when comparing between male and female mice on 60% HFD diet.

**Figure 4:**
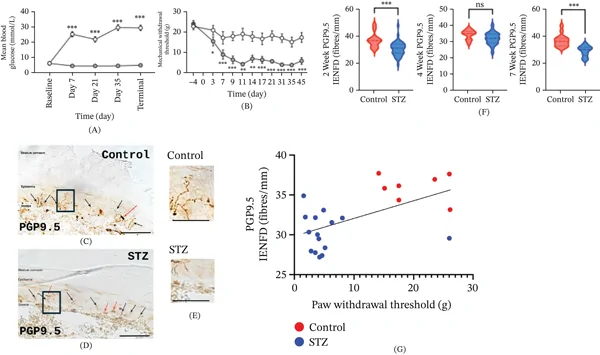
Type 1 STZ‐induced diabetic sensory neuropathy with reduced intraepidermal nerve fibre staining. Streptozotocin (STZ)‐induced Type 1 diabetic neuropathic pain led to (A) increased blood glucose 7 days following STZ injection and this remained for the remainder of the study when compared with sham control rats. (B) There was a reduction in mechanical withdrawal thresholds in the STZ‐treated group when compared with sham controls, this was demonstrated from Day 3 postinjection and remained for the duration of the study (two‐way ANOVA with post‐Bonferroni test,  ^∗∗∗^ *p* < 0.001, ns = not significant, *n* = 11 control and *n* = 15 STZ per group). Representative images of normal innervation of hind paw footpad in from (C) age‐matched control and (D) STZ‐treated male Wistar Han ISG rat stained with anti‐PGP 9.5 antibody (Scale bar = 10 * μ*m), (E) with higher power images highlighted in black boxes (Scale bar = 5 * μ*m). (F) Innervation of plantar hind paw footpad in diabetic male Wistar Han ISG Rat 2 and 7 weeks after STZ administration demonstrated reductions in IENF versus age matched control. (G) This reduction was correlated with reductions in nociceptive mechanical withdrawal threshold. Black arrows indicate intraepidermal nerve fibres, and red arrow indicates PGP 9.5‐positive Langerhans cells (unpaired *t* test,  ^∗∗∗^ *p* < 0.001, ns = not significant, *n* = 7 control and *n* = 15 STZ). Scale bar = 20 * μ*m.

**Figure 5:**
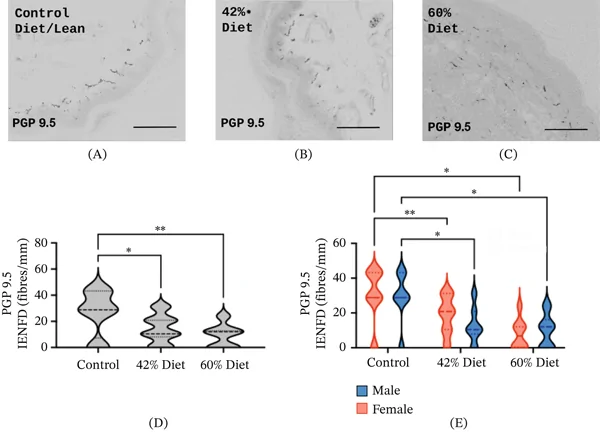
Dietary induced sensory neuropathy with reduced intraepidermal nerve fibre staining across sexes. Representative images of innervation of hind paw footpad from (A) age‐matched lean control diet, (B) 42% fat diet and (C) 60% fat diet at 12 weeks, with anti‐PGP 9.5 antibody black arrows indicate intraepidermal nerve fibres and red arrows indicate PGP 9.5‐positive Langerhans cells. (D) Innervation of plantar hind paw footpad in 42% fat diet and 60% fat diet mice demonstrated reductions in IENF versus lean age matched control, (E) which was also presented in females but only 60% fat diet led to reductions in IENF in male mice (one‐way ANOVA, two‐way ANOVA with post‐Bonferroni  ^∗^ *p* < 0.05,  ^∗∗^ *p* < 0.01 and  ^∗∗∗^ *p* < 0.001, ns = not significant, *n* = 6 per group). Scale bar = 10 * μ*m.

**Figure 6:**
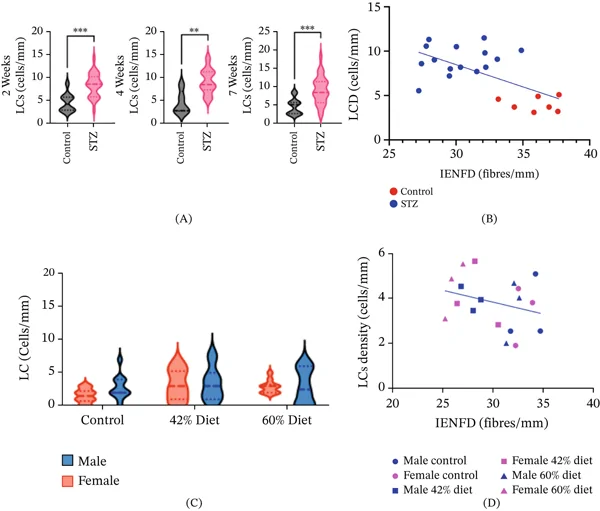
Increased Langerhan cell density in Type 1 STZ‐induced diabetes is associated with mechanical allodynia. (A) Langerhans cell density was increased in STZ‐induced Type‐1 diabetic rats at 2, 4 and 7 weeks after STZ administration versus age‐matched sham control. (B) There was a negative correlation between PGP9.5 intraepidermal nerve fibre density and Langerhans cell density in age‐matched sham control and STZ‐treated rats (*r* = −0.6214, *p* = <0.002, *n* = 7 control, *n* = 15 STZ). (C) Langerhans cell density did not change between male or female mice fed for 12 weeks on control chow, 42% fat diet or 60% fat diet. (D) There was no correlation between intraepidermal nerve fibre density and Langerhans cell density in male and female fed control, 42% HFD or 60% HFD mice (*p* = 0.255, *R* ^2^ = 0.079). (Unpaired *t* test, two tailed:  ^∗∗∗^ *p* < 0.001; no significant difference was determined using two‐way ANOVA, *n* = 6).

In the STZ‐induced Type 1 diabetes model, a fourfold increase in blood glucose (Figure 4A, > 16 mmol/L) and mechanical allodynia developed in STZ treated rats from Day 7 where mechanical withdrawal thresholds were significantly lower in STZ animals than control animals and remained significantly lower throughout the 7‐week study (Figure 4B). IENFD (PGP 9.5 positive IENFD, Figure 4C–E in plantar skin was significantly reduced compared with controls at 2 and 7 weeks (*p* < 0.001, Figure 4F), which was positively correlated to mechanical withdrawal threshold (*r* = 0.57, *p* < 0.005, Figure 4G). The 42% and 60% diet both induced labelled PGP9.5 IENFD reductions (Figure 5A–C) compared with mice fed control diet (Figure 5D). Additionally, mice fed the 60% HFD had significantly lower IENFD in males and females compared with the control diet (Figure 5E). The 42% HFD group also showed significant reductions in females (*p* = 0.002) but not males (Figure 5E). LC number in STZ hind paw skin was significantly higher than controls at all time points (2, 4 and 7 weeks; *p* < 0.001, Figure 6A). LC expression remained consistent across time in both groups, suggesting early and sustained immune involvement in diabetic skin, with a strong negative correlation between PGP9.5 IENF and LC number (Figure 6B). There were no increases in LC number in experimental diet HFD‐fed mice and no correlation between PGP9.5 IENF and LC number (Figure 6C,D).

**Figure 7:**
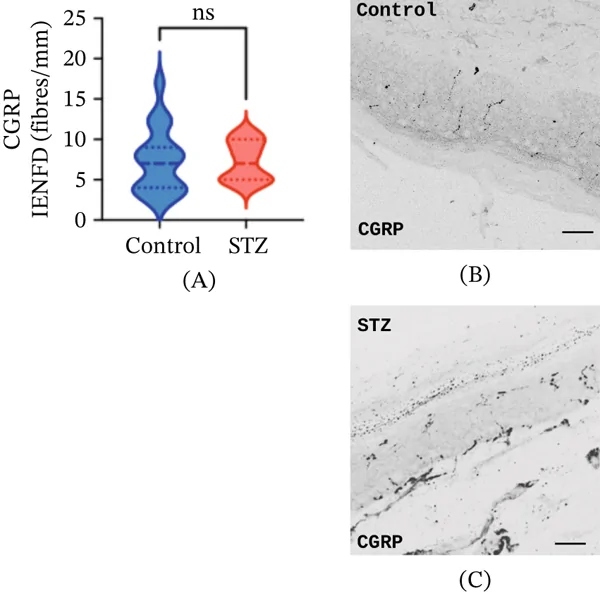
Preservation of nociceptor intraepidermal nerve density across sexes in Type 1 STZ‐induced diabetes and dietary induced sensory neuropathy. (A) There was no change in CGRP IENF innervation of plantar hind paw footpad in diabetic male Wistar Han ISG rat at 2, 4 or 7 weeks after STZ administration versus age‐matched control. Representative images of CGRP innervation of hind paw footpad in (B) age‐matched control and (C) STZ‐induced Type 1 diabetic. Black arrows indicate CGRP intraepidermal nerve fibres (unpaired *t* test, ns = not significant, *n* = 11 control, *n* = 14 STZ). Scale bar = 20 * μ*m.

**Figure 8:**
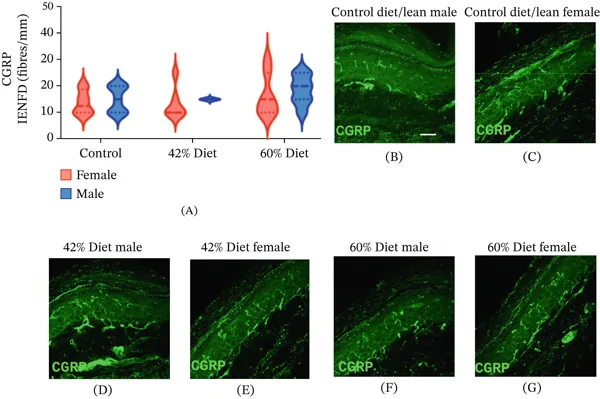
Preservation of nociceptor intraepidermal nerve density in dietary induced sensory neuropathy. (A) In age‐matched lean control diet, 42% fat diet and 60% fat diet at 12 weeks, there was no change in CGRP IENF innervation. (B–G) Representative images of CGRP innervation of hind paw footpad in age‐matched lean control diet, (B) 42% fat diet and (C) 60% fat diet at 12 weeks, with high magnification inset (two‐way ANOVA, ns = not significant, *n* = 6 per group). Scale bar = 20 * μ*m.

To explore nociceptor‐specific markers, calcitonin gene‐related peptide (CGRP) expression was quantified (Figure 7A,B). In STZ‐treated diabetic rats, total CGRP‐reactive fibre density did not differ significantly from controls at Week 7 (Figure 7A). In both HFD‐fed experimental groups versus control diet, CGRP fibre density showed no significant differences (Figure 8A–G). Additionally, there was no difference in CGRP IENFD when sex was considered across differing experimental dietary interventions.

## 4. Discussion

The emergence of chronic pain in diabetic neuropathy presents a fundamental clinical phenotype. Despite the progressive degeneration of peripheral sensory nerves, a defining feature of diabetic sensory neuropathy, many patients report heightened pain sensitivity. Unfortunately, there is limited effective analgesia available to treat these individuals. In relation to rodent models, classification of sensory neuropathy is depicted as nociceptive behavioural readouts alongside traditional histopathological assessments [23]. This includes diabetic sensory neuropathy defined in part by the total loss of IENFs, interpreted as peripheral sensory nervous system (PNS) degeneration, which is present in patients [24] and experimental rodent models [22, 25]. However, our data suggest that the decline in IENF density is not homogeneous across the sensory neuron population. Rather than all nerve fibres being equally affected, there is a preservation of CGRP‐expressing fibres in diabetic skin. Here, diabetic neuropathic pain presents alongside global small fibre degeneration, whereas our data highlight a more complex model where selective sparing of a distinct nociceptor population drives the pain phenotype. In this study, we explored this hypothesis in a rodent model of Type 1 and Type 2 diabetic painful sensory neuropathy, focusing on the structural and functional characteristics of CGRP‐positive IENFs, with implications for the mechanisms underlying diabetic pain.

Identification of IENF inherently relies upon total nerve fibre skin innervation using panneuronal marker PGP 9.5, which is utilised to demonstrate degeneration of the PNS. However, further understanding of nerve fibre loss in diabetes is not a uniform phenomenon but one that may spare specific nociceptor subtypes specifically associated with nociception and pain perception. Here, we present that peptidergic nociceptor afferents expressing CGRP are preserved in Type 1 and prediabetes/Type 2 diabetic sensory neuropathy. This supports the ideology that pain manifests and is persistent, despite overall denervation. Few studies in human [26, 27] or rodent [28] models have explored CGRP IENF in diabetic sensory neuropathy. Clinical observations align with ours, with studies involving skin biopsy from patients with painful diabetic neuropathy revealing preservation of neuropeptides such as CGRP and substance P [26], despite demonstrated reductions in total IENF numbers in human diabetic sensory neuropathy patients [26]. However, to note, recent observations in alternative studies demonstrate minimal association between painful phenotype in patients and/or potential reductions of CGRP positive IENF in diabetes [27, 29, 30]. However, this CGRP reduction was principally observed in bone and pancreas [29, 30], which are distinct to skin with reference to CGRP nerve density. In addition, in diabetic human patients, there is no reference whether patients with reduced CGRP in the skin also presented other markers or history of sensory neuropathy or disturbance in pain. CGRP is well characterised and associated with increased nociceptor excitability and spontaneous activity of surviving nociceptors [31]. Preservation and potential damage to CGRP nociceptors can underpin chronic pain states, as well as these sensory neuronal maladaptations inducing cognitive disturbances including anxiety and sleep deprivation [32], which are present in diabetic painful sensory neuropathy patients [33] and are important diagnostic indicators for neuropathy score [34, 35]. Conversely, patients with painless diabetic neuropathy tend to exhibit a more uniform loss of both peptidergic and nonpeptidergic fibres, highlighting a mechanistic divergence between painful and unpainful phenotypes [9]. Our preclinical findings in these rodent models mirror this clinical pattern and affirm the translational relevance of CGRP‐positive fibre preservation as both a biomarker and potential therapeutic target as well as demonstrating the translational strength of rodent diabetic sensory neuropathy models. However, it must be noted that in this study diabetic models incorporate both rats and mice. These were performed in line with sector guidelines, as Type 1 rodent models are typically performed using rats with numerous articles outlining guidance for this model [22]. For mouse, however, it is difficult to induce Type 1 diabetes because of resistance of mice to STZ exposure, leading to inconsistent alterations in metabolic phenotyping or alternatively high lethality rates [36]. Additionally, sex is an important biological variable in the manifestation and progression of chronic pain states [37], with clinical and preclinical evidence demonstrating that females exhibit greater sensitivity to noxious stimuli and an elevated prevalence of chronic pain conditions compared with males. In rodent models, sex differences in nociceptive processing have been attributed to divergent neuroimmune mechanisms principally centrally via hormonal and/or inflammation, with microglia‐dependent pathways predominating in males and T‐cell mediated mechanisms playing a greater role in females [38]. This is further evidenced with minimal difference in the nociceptor profile across sex groups [39]. In the context of diabetic sensory neuropathy, these sex‐specific differences may further modulate the peptidergic fibre response acting centrally for perception of pain rather than identification of tissue damaging stimuli. This CNS component potentially contributes to the differential nociceptive phenotypes observed between males and females in the present study. The interplay between sex hormones, immune signalling and nociceptor plasticity therefore represents an important avenue for future investigation, particularly in diet‐induced models and may have meaningful implications for the development of sex‐informed therapeutic strategies targeting CGRP‐positive afferents in painful diabetic neuropathy.

In our rodent models of diabetic sensory neuropathy, mechanical allodynia appeared early and was sustained over several weeks, indicating stable hypersensitivity, which returned to baseline over time following experimental dietary intervention, indicative of the clinical setting, with many patients experiencing loss of sensation. Additionally, dietary induced models demonstrate a persistent and robust heat hypersensitivity for the duration of the study, similar to our previous rat Type 1 models [40]. Concurrently, total IENF density declined progressively, reflecting sensory nerve terminal loss. However, CGRP‐positive fibres remained consistently present. This finding suggests that peptidergic nociceptors are either more resilient to diabetic injury or are maintained through compensatory mechanisms, possibly via local inflammatory or metabolic cues. Their selective survival provides a plausible substrate for persistent nociceptive signalling, even when the broader sensory landscape is deteriorating. Additionally, CGRP is not merely a marker of peptidergic identity; it is a potent neuropeptide involved in nociceptive transmission, neurogenic inflammation and peripheral sensitisation. The continued presence in diabetic skin suggests an active functional role in sustaining pain signalling, rather than reflecting passive structural survival. These CGRP‐positive peptidergic nociceptor terminals are likely to remain responsive to noxious thermal and chemical stimuli, aligning with behavioural evidence of evoked hypersensitivity. Thus, in diabetic animals, the persistent presence of CGRP within the epidermis is indicative of ongoing nociceptor activity and supports the view that a subpopulation of sensory afferents becomes maladaptively preserved and hyperactive. Here, it therefore needs to be considered that ectopic or spontaneous nociceptor discharge has been demonstrated to drive chronic pain states [41], which acts as a potential avenue by which diabetic painful sensory neuropathy is driven [41]. This neuronal electrophysiological event additionally may not develop from intact but from damaged nociceptors. Therefore, the lost PGP9.5 population could underpin the driven pain state [42]. However, this phenomenon relies upon denervation, and here, we propose that evoked nociceptive behaviours require application and recognition of a stimulus to evoke nociceptor activity to induce rodent nociceptive withdrawals. This is an area of study that we have not continued to explore, though nociceptor ablation has been performed by others to evaluate this concept [32].

Preservation of CGRP IENF may, in part, rely upon an immune‐sensory interaction, a feature identified in painful diabetic neuropathy [26]. As total fibre loss progresses, CGRP‐expressing fibres are preserved because of neuroprotective capacity of the local immune response, whilst also driving hyperexcitability, sustaining pain through ongoing interaction with secreted pro‐inflammatory mediators such as nerve growth factor a potent nociceptor sensitiser and neuroprotector. This preservation of CGRP‐positive nociceptors is supported in Type 1 diabetes models where local interactions with the immune system, particularly LCs, specialised antigen‐presenting cells in the epidermis. However, this would be restricted to Type 1 diabetes. In our study, STZ‐treated diabetic animals displayed mechanical allodynia and a significant increase in LC density in diabetic skin, although total IENF counts declined, a correlation previously observed in painful diabetic neuropathy patients and in diabetic human corneal nerve damage [43, 44]. Interestingly, CGRP‐positive fibres were not reduced to the same extent, implying a selective sparing of these nociceptors. This inverse correlation between LCs and total IENFs raises the possibility that immune‐mediated processes may drive degeneration in a fibre‐specific manner. Though this was not replicated in the dietary intervention model, metabolic disease has been showcased to induce pro‐inflammatory systemic profile. LCs are known to release both neurotrophic and inflammatory factors and could exert opposing effects on different fibre populations to promote survival or regeneration in some whilst contributing to degeneration in others. The preferential maintenance of CGRP‐positive fibres in this immunologically active environment suggests that these nociceptor afferents are protected or even activated by inflammatory signals. In contrast, in the HFD model, male and female C57Bl/6 mice were fed either control chow, 42% fat or 60% fat diets for 12 weeks; mechanical and thermal hypersensitivity developed in HFD‐fed mice in the absence of local inflammatory activity through resident cells. Importantly, IENFD declined significantly in 60% HFD mice, particularly in females, suggesting that metabolic dysregulation alone is sufficient to impair peripheral innervation. However, LC density changes did not correlate significantly with IENFD, indicating a more complex or delayed immune response in diet‐induced neuropathy, whereas CGRP IENF was preserved.

In conclusion, this work highlights the selective preservation of CGRP‐expressing nociceptors in Type 1 and 2 diabetic sensory neuropathy as a key marker of diabetic painful sensory neuropathy. The differential patterns observed of Type‐1 and Type‐2 rodent models of diabetes provide further understanding of sensory diabetic neuropathy, exploring functional subtypes of surviving fibres rather than total fibre loss alone. By incorporating both structural and neurochemical data for consideration of peptidergic nociceptor subtypes and particularly CGRP, future research can better identify targets for therapeutic intervention and pave the way for more effective, personalised treatments for diabetic neuropathic pain.

## Author Contributions

**Lisa A. Lione:** conceptualization, methodology, formal analysis, supervision, funding acquisition, project administration, writing – review and editing. **Lydia D. Hardowar:** investigation, methodology, formal analysis, data curation, validation, visualisation, writing – original draft, writing – review and editing. **Michael T. Lanigan:** investigation, methodology, formal analysis, validation, writing – review and editing. **Jessica Bates:** investigation, methodology, formal analysis, resources, writing – review and editing. **William Blackstone-Whines:** investigation, methodology, formal analysis, writing – review and editing. **Harry Jones:** investigation, methodology, formal analysis, data curation, writing – review and editing. **Richard P. Hulse:** conceptualization, methodology, formal analysis, supervision, funding acquisition, writing – original draft; writing – review and editing.

## Funding

This study was funded by the EFSD/Boehringer Ingelheim European Research Programme in Microvascular Complications of Diabetes (BI18_5), Diabetes Research and Wellness Foundation, Society for Endocrinology and Nottingham Trent University.

## Disclosure

All authors have read and approved the final version of the manuscript. Lisa A. Lione, Lydia D. Hardowar, Michael T. Lanigan, Jessica Bates, William Blackstone‐Whines, Harry Jones and Richard P. Hulse had full access to all of the data in this study and takes complete responsibility for the integrity of the data and the accuracy of the data analysis. All authors have provided written permission to be authors in accordance with ICMJE recommendations. None of the cited references have been retracted.

## Conflicts of Interest

The authors declare no conflicts of interest.

## Supporting information


**Supporting Information** Additional supporting information can be found online in the Supporting Information section. Figure S1: Dietary induced neuropathic pain in males. Male C57 BI6 mice were fed either control chow (lean; *n* = 28), 42% fat diet (*n* = 6) or 60% fat diet (*n* = 28). (A) Fasted blood glucose and (B) body weight were recorded regularly during the study, with 60% HFD dietary intervention inducing elevations in fasted blood glucose and body weight. (C) Mechanical nociceptive withdrawal threshold was reduced initially following 60% HFD dietary intervention that later resolved compared with lean control group. (D) Heat nociceptive withdrawal latency was measured using Hargreaves test demonstrating a robust reduction following 60% HFD dietary intervention, with 42% HFD inducing partial hypersensitivity (two‐way ANOVA with mixed effects and multiple comparisons,  ^∗^ *p* < 0.05,  ^∗∗^ *p* < 0.01 and  ^∗∗∗^ *p* < 0.001 = lean vs. 60% diet, *p* < 0.05 = lean vs. 42% diet). Figure S2: Dietary induced neuropathic pain in females. Female C57 BI6 mice were fed either control chow (lean; *n* = 6), 42% fat diet (*n* = 6) or 60% fat diet (*n* = 6). (A) Fasted blood glucose and (B) body weight were recorded regularly during the study, with 60% HFD dietary intervention inducing elevations in fasted blood glucose and body weight. (C) Mechanical nociceptive withdrawal threshold were reduced compared with lean control group, with this occurring at later stages of the study beyond 10 weeks. (D) Heat nociceptive withdrawal latency was measured using Hargreaves test demonstrating a robust reduction following 60% HFD dietary intervention (two‐way ANOVA with mixed effects and multiple comparisons,  ^∗^ *p* < 0.05 and  ^∗∗^ *p* < 0.01 = lean vs. 60% diet).

## Data Availability

The data that support the findings of this study are available from the corresponding author upon reasonable request.
